# The Effect of Acupuncture on Pain, Prostaglandin E2, and Interleukin‐6 in Septorhinoplasty Operations: A Randomized Clinical Trial

**DOI:** 10.1155/anrp/7816719

**Published:** 2025-12-07

**Authors:** Yavuz Orak, Filiz Alkan Baylan, Fatma Bilgen, Filiz Orak, Alper Ural, Sedat Yildiz, Saime Sagiroglu, Harun Karaduman, Adem Doganer

**Affiliations:** ^1^ Faculty of Medicine, Department of Anesthesiology and Reanimation, Kahramanmaras Sutcu Imam University, Kahramanmaras, Türkiye, ksu.edu.tr; ^2^ Faculty of Medicine, Department of Biochemistry, Kahramanmaras Sutcu Imam University, Kahramanmaras, Türkiye, ksu.edu.tr; ^3^ Department of Plastic, Reconstructive and Aesthetic Surgery, Medilife Health Group, Istanbul, Türkiye; ^4^ Faculty of Medicine, Department of Microbiology, Kahramanmaras Sutcu Imam University, Kahramanmaras, Türkiye, ksu.edu.tr; ^5^ Department of Plastic, Reconstructive and Aesthetic Surgery, Memorial Ankara Hospital, Ankara, Türkiye, memorial.com.tr; ^6^ Physical Medicine and Rehabilitation Department, Private Doctor’s Office, Isparta, Türkiye; ^7^ Faculty of Medicine, Department of Otorhinolaryngology, Kahramanmaras Sutcu Imam University, Kahramanmaras, Türkiye, ksu.edu.tr; ^8^ Faculty of Medicine, Department of Plastic, Reconstructive and Aesthetic Surgery, Kahramanmaras Sutcu Imam University, Kahramanmaras, Türkiye, ksu.edu.tr; ^9^ Faculty of Medicine, Department of Biostatistics and Medical Informatics, Kahramanmaras Sutcu Imam University, Kahramanmaras, Türkiye, ksu.edu.tr

## Abstract

**Objective:**

The purpose of this study was to investigate the effects of acupuncture on pain, prostaglandin E2 (PGE2), and interleukin‐6 (IL‐6) levels during septorhinoplasty surgeries.

**Materials and Methods:**

This randomized, controlled study included 70 patients. The patients were divided into two groups: an acupuncture group (*n* = 35) and a control group (*n* = 35). The acupuncture group received bilateral press needle acupuncture at the PC 6 and ST 36 points 24 h before surgery. Blood samples were collected for analysis and comparison of preoperative and postoperative levels of IL‐6 and PGE2. The primary outcomes were the postoperative visual analog scale (VAS) scores.

**Results:**

In terms of evaluating postoperative pain, no statistically significant differences were observed between the study groups with regard to VAS scores. At 30 min after surgery, fewer patients in the acupuncture group needed analgesics than in the control group (*p* = 0.044). Postoperative IL‐6 levels were lower in the acupuncture group than in the control group (*p* = 0.014). There was no significant difference in postoperative PGE2 levels between the groups (*p* = 0.568). The acupuncture group had lower diastolic blood pressure (DBP) and mean arterial blood pressure (MAP) at 30 min intraoperatively and lower DBP at 60 min (*p* = 0.012, *p* = 0.026, and *p* = 0.012, respectively). At 15 min into the operation, the heart rate was higher in the acupuncture group than in the control group (*p* = 0.039). After surgery, the acupuncture group had lower blood pressure at 5 min and 6 h after surgery than the control group (*p* = 0.034 and *p* = 0.041, respectively).

**Conclusions:**

The evidence from this study suggests that acupuncture can reduce the need for pain medication after septorhinoplasty surgery, and by decreasing IL‐6 levels, it may contribute to the inflammatory process.

**Trial Registration:**

ClinicalTrials.gov: NCT04009070

**Summary**

• The goal of employing press needle acupuncture in septorhinoplasty surgeries is to avoid postoperative pain, swelling, and nausea or vomiting.
• Using press needle acupuncture during septorhinoplasty surgeries resulted in fewer patients requiring analgesics after surgery.
• The acupuncture was effective in reducing the postoperative IL‐6 level.
• Our study will pave the way for future research on the use of acupuncture in septorhinoplasty surgeries.

## 1. Introduction

Acupuncture is an ancient therapeutic technique that has been used to treat a variety of medical conditions, including pain, musculoskeletal diseases, inflammation [1, 2], osteoarthritis of the knee [3], chronic headaches, and shoulder pain [4]. The analgesic effect of acupuncture is attributable to the activation of acupuncture points. Acupuncture has been demonstrated to stimulate neural pathways that modulate pain and inflammatory responses [5]. The efficacy of acupuncture in reducing pain following nasal septoplasty [6] has been demonstrated, and the application of acupressure in patients experiencing labor pains resulted in a reduction in the intensity of labor pains [7].

Septorhinoplasty is a surgical procedure that is frequently performed by plastic surgeons and otolaryngologists. Postoperative comfort is of equal importance to the success of the operation, particularly in the context of cosmetic surgery. It has been demonstrated that rhinoplasty is a more painful procedure than septoplasty. It is well established that any operation on the external nose and nasal skeleton can be substantially painful, yet there is a paucity of prospective studies evaluating early pain after rhinoplasty or septorhinoplasty [8].

Opioid agents are commonly used as postoperative analgesics in clinical practice, but they tend to cause dose‐dependent adverse reactions. These include gastrointestinal reactions, respiratory depression, urinary retention and pruritus [9]. Postoperative pain is a type of acute inflammatory pain that occurs after a surgical operation and subsides once the tissue has healed [10]. Prostanoids are widely considered to play a role in various physiological processes, including pain perception, fever induction, edema, and inflammation. It is evident that acupuncture exerts an anti‐inflammatory effect by affecting the hypothalamic–pituitary adrenal (HPA) axis, thereby reducing cyclooxygenase‐2 (COX‐2) and prostaglandin E2 (PGE2) levels. Acupuncture also activates the sympathetic nervous system and causes the body to release opioids [11]. PGE2 has been identified as the fundamental proinflammatory prostanoid, playing a pivotal role in nociceptive processing and sensitization within the peripheral nervous system [12]. PGE2 is well known to be a key mediator of inflammation, contributing to the pain associated with inflammation and mechanical injury [13]. Surgery causes tissue damage, which triggers a systemic response involving proinflammatory modulators. PGE2 is the predominant eicosanoid that is released following surgical procedures and is associated with inflammatory responses, elevated body temperature, and discomfort [14]. PGE2 and interleukin‐6 (IL‐6) are important components of the inflammatory response associated with surgery in patients and may affect clinical outcomes [15]. IL‐6 has been identified as a sensitive indicator of tissue injury, with levels of serum enhancement exhibiting a direct correlation with the extent of surgical intervention [16].

**Figure 1:**
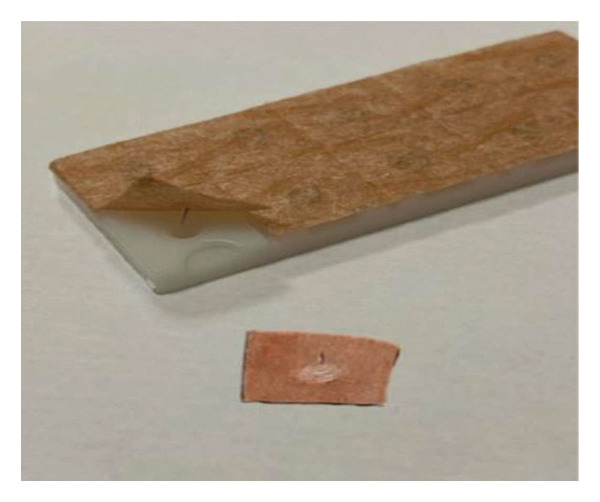
Press needle [17].

Press needles (PNs) represent a distinct category of acupuncture needles (Figure 1) that have been developed from traditional intradermal needles utilized in Japan. PN has been demonstrated to provide sustained stimulation of targeted acupuncture points in a safe and noninvasive manner for a period of several days [18]. PN stimulates acupuncture points and regulates the body’s nervous and endocrine systems. The PN needle is small and thin, which can relieve pain and extend the therapeutic effect by holding the needle for a longer time. Consequently, it exhibits a reduced traumatic effect, is more acceptable to patients, and is more appropriate for clinical application [19].

The present study investigates the effects of PN acupuncture application on pain relief and the measurement of PGE2 and IL‐6 levels in septorhinoplasty surgery.

## 2. Methods

### 2.1. Experimental Design and Patients

This randomized controlled trial was conducted at Kahramanmaras Sutcu Imam University between August 2019 and April 2020. The study received ethical approval from Kahramanmaras Sutcu Imam University, Faculty of Medicine, Ethics Committee for Clinical Research, on July 13, 2018 (Approval Number: 2018/12‐17), as well as from the Turkish Ministry of Health (MoH) on November 23, 2018 (Approval Number: 77979112). Written consent was obtained from each participant. Participants were randomly divided into two groups: an acupuncture group (*n* = 35) and a control group (*n* = 35). The acupuncture treatment was administered in the preoperative preparation room by the project manager physician, who had attended and successfully completed a Ministry of Health (MoH)‐certified acupuncture training program for physicians at Ankara Yıldırım Beyazit University, Faculty of Medicine, Traditional and Complementary Medicine Center.

The inclusion criteria for this study are as follows: Patients between the ages of 18 and 45 who are classified as American Society of Anesthesiologists (ASA) Class I or Class II have undergone septorhinoplasty. All patients voluntarily participated in the study.

Patients were excluded for the following reasons: chronic inflammatory disease, systemic or local infection, anticoagulation therapy, central nervous system disorders, allergies to local anesthetics and painkillers, spinal deformities, or cognitive dysfunction.

### 2.2. Intervention

The study’s sample population comprised 70 patients. Patients were divided into two groups: an acupuncture group (*n* = 35) and a control group (*n* = 35). The acupuncture group was medicated with bilateral PN (Figure 1, Hua Long, China, 0.22 × 1.5 mm) acupuncture 24 h prior to surgery at acupuncture points PC 6 (Neiguan, located on the inner arm, approximately 2 cm over the midline of the wrist crease) and ST 36 (Zusan Li, approximately 1–2 cm lateral to the tuberositas tibiae). The control group did not receive acupuncture. On the day of the surgical procedure, blood samples were obtained from both groups in the preoperative room via peripheral venous access for the purpose of analyzing IL‐6 and PGE2 levels. The patients were subsequently transferred to the surgical suite for the purpose of monitoring vital signs and acquiring electrocardiographic data. The following parameters were monitored: systolic blood pressure (SBP), diastolic blood pressure (DBP), mean arterial blood pressure (MAP), peripheral oxygen saturation (SpO2), and electrocardiography (ECG) (Dräger infinity kappa, Dräger Medical GmbH Lübeck, Germany). The induction of anesthesia was facilitated by sodium thiopental (I.E. Ulagay İlaç Sanayi Türk A.Ş., İstanbul) at 5 mg/kg, fentanyl (TalinatR, Vem, Istanbul, Türkiye) at 1 mcg/kg, and rocuronium (MyocronR, Vem, Istanbul, Turkey) at 0.6 mg/kg via intravenous injection. Sevoflurane (SevoraneR, AbbVie, Istanbul, Turkey) at a concentration ranging from 1% to 2% MAC, in conjunction with a 50% oxygen (O_2_) mixture, was utilized to maintain anesthesia. The primus anesthesia machine (Dräger, Lübeck, Germany) was utilized for the intraoperative maintenance of anesthesia. A variety of parameters were documented, including patient age (years), sex, body mass index (BMI, m2/kg), ASA score, smoking status, extubation time (minutes), duration of operation (minutes), tramadol (Abdi Ibrahim İlaç San. Ve Tic. A.Ş. Istanbul, Turkey) dosage (mg), and the number of patients requiring analgesics after 30 min from the surgical operation. Additionally, the necessity for an additional dose of paracetamol after surgery was recorded. Following the implementation of intubation in the patients, intraoperative measurements of MAP, SBP, DBP, HR, and SpO_2_ were recorded at 5‐, 15‐, 30‐, 60‐, and 120‐min intervals, respectively. For the procedure of extubation, atropine sulfate (0.02–0.06 mg per kilogram, supplied by Drogsan İlaçları San. ve Tic. A.Ş., Turkey) and neostigmine methylsulfate (0.05 mg per kilogram, supplied by Adeka İlaç Sanayi ve Ticaret A.Ş., Turkey) were utilized to neutralize the effects of muscle relaxants. In order to achieve postoperative analgesia, patients were administered 1 mg/kg of intravenous standard tramadol 20–30 min before the conclusion of the surgical intervention. Following the conclusion of the surgical intervention, the administration of anesthetic gases was terminated, and the patients were provided with ventilation using a mask containing 100% oxygen until they regained consciousness. The extubation of patients was contingent upon the presence of SpO_2_ values exceeding 97%, in conjunction with adequate respiratory effort. Postoperative follow‐up was performed on the operating table at the 5th min, in the postoperative recovery room at the 30th min, and in the plastic surgery ward at the 60th min, and 3–6–12–24‐h results were recorded. The pain condition was measured by using the visual analog scale (VAS) score after patients were informed how to use the VAS (VAS: a 10‐mm horizontal crease presenting a point with the following ranges: 0, No Pain; and 10, the Worst Pain Devisable). In the postanesthesia care unit, patients were asked about their VAS score. If the VAS score was 4 or higher, 1 g of paracetamol was administered intravenously. In the acupuncture group, PNs were retained for 24 h postsurgery. The patients were instructed to stimulate these acupuncture points on a regular basis. Blood samples were aggregated to measure PGE2 and IL‐6 levels at 24 h after surgery. Postoperative and preoperative IL‐6 and PGE2 levels were compared between groups.

### 2.3. Study Outcomes

#### 2.3.1. Primary Outcomes

Primary outcome was the VAS score at 5–30–60 minutes and 3–6–12–24 hours postoperatively.

#### 2.3.2. Secondary Outcomes

PGE2 and IL‐6 levels at 24 h and MAP, SBP, DBP, HR, and SpO_2_ levels at 5–15–30–60 and 120 min intraoperatively and at 5–30–60 minutes and 3–6–12–24 hours postoperatively were secondary outcomes. Nausea and vomiting at 3–6–12–24 h postoperatively were also our secondary outcomes.

### 2.4. Sample Size Calculation

The sample size calculation was determined using power analysis: Type I error *α*: 0.05, Type II error β: 0.20, power: 0.80 [1]. The minimum size for each group was 34 (*n* = 34) based on the calculation of statistical parameters for the VAS score variable at 2 h with Group 1: 46 ± 15 and Group 2: 60 ± 24.

### 2.5. Randomization and Blinding

Patients were randomly divided into two groups: an acupuncture group (*n* = 35) and a control group (*n* = 35). This randomization was conducted using internet‐based randomization software. The participants were randomly assigned to the groups with a 1:1 ratio. The nurses who monitored the postoperative outcomes in the postoperative unit and the plastic surgery service, as well as the anesthesiologists who closely observed the patients throughout the surgical procedures, were unaware of the study’s objectives.

### 2.6. Points of Acupuncture (Figure 2)

ST 36 (Zusan Li) is located on the anterior lower leg, one‐finger width (third finger) from the tibia anterior crest. PC 6 (Neiguan) is located on the forearm, palmar aspect, 2 cun above the wrist transverse crease [20]. Cun is a traditional Chinese unit of measurement for length. The interphalangeal joint breadth of the first finger is 1 cun [21].

**Figure 2:**
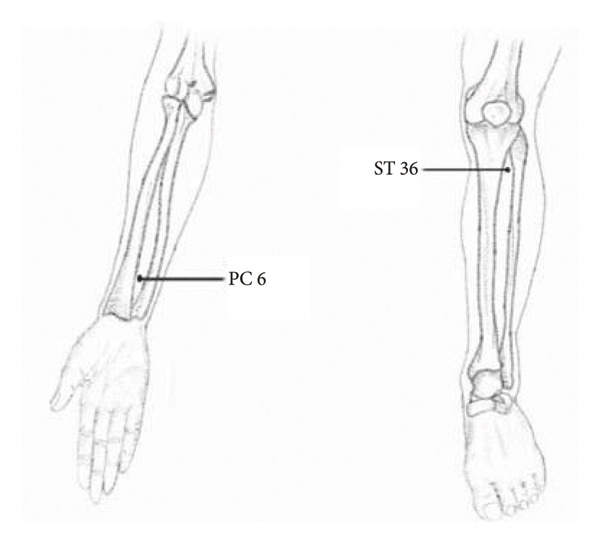
Locations of acupuncture points.

### 2.7. Biochemical Analysis

Human IL‐6 and PGE2 serum levels were analyzed with an ELISA kit (Shanghai Sunred Biological Technology Co., Ltd), an automatic ELISA reader (Thermo Scientific, Finland), and a computer program (Scanlt for Multiskan FC 2.5.1). For IL‐6 (Catalog No: CK‐bio‐12137), the sensitivity was 10 pg/mL, and the assay range was 15–800 pg/mL. For PGE2 (Catalog CK‐bio‐13061), the sensitivity was 10 pg/mL, and the assay range was 100–2000 pg/mL. The measurement of results was recorded in units of pg/mL.

### 2.8. Statistical Analysis

The Shapiro–Wilk test was employed to assess the normality distribution of the data. In instances where the variables were distributed normally, the independent samples *t* test was employed to facilitate a comparison between independent groups. The difference between the measurements taken before and after the intervention was evaluated using the paired *t* test. The discrepancy in distribution between categorical variables was gauged employing both the chi‐square and Fisher’s exact test methodologies. Statistical parameters were expressed as mean ± SD and *n* (%). The statistical significance was set at *p* < 0.05. The IBM SPSS Statistics version 22 (IBM SPSS for Windows version 22, IBM Corporation, Armonk, New York, USA) was utilized for the analysis of the article’s data.

## 3. Results

**Figure 3:**
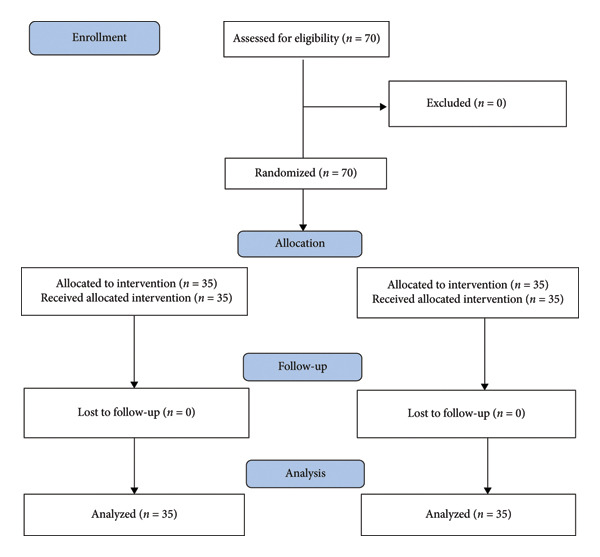
Consort diagram of the study.

The study group comprised 70 patients (Figure 3). The mean age of the acupuncture group was 23.36 years (±6.93 years), and the mean age of the control group was 26.31 years (±8.26 years). A subsequent analysis revealed no statistically significant differences between the groups in terms of demographic or baseline variables. As indicated by the results presented in Table 1, at 30 min after surgery, a lower percentage of patients in the acupuncture group required postoperative analgesics in comparison to the control group (*p* = 0.044).

**Table 1:** The demographic and clinical data comparison between the acupuncture and control groups.

		Acupuncture (*n* = 35)	Control (*n* = 35)	MD (CI)	ES (CI)	*p*
Age (years)	Mean ± SD	23.36 ± 6.93	26.31 ± 8.26	−2.95 (−6.59, 0.69)	−0.39 (−0.86, 0.09)	0.115

Sex	Female, *n* (%)	21 (60)	19 (54.30)			0.629
	Male, *n* (%)	14 (40)	16 (45.70)			

ASA	1	35 (100)	35 (100)			—

BMI (m^2^/kg)	Mean ± SD	21.27 ± 3.35	22.46 ± 3.03	−1.19 (−2.71, 0.33)	−0.37 (−0.84, 0.10)	0.131

Smoking	No, *n* (%)	28 (80)	25 (71.43)			0.403
	Yes, *n* (%)	7 (20)	10 (28.57)			

Time to extubation (min)	Mean ± SD	6.97 ± 3.19	8.91 ± 5.11	−1.94 (−3.97, 0.09)	−0.46 (−0.92, 0.02)	0.061

Duration of operation (min)	Mean ± SD	95.57 ± 44.91	85.57 ± 34.34	10.00 (−9.07.29.07)	0.25 (−0.22, 0.72)	0.299

Tramadol dose (mg)	Mean ± SD	63.71 ± 1.07	64.46 ± 16.37	−0.75 (−6.28, 4.78)	−0.06 (−0.53, 0.40)	0.825

ARPO 30 min	No, *n* (%)	16 (45.71)	8 (22.86)			0.044^∗^
	Yes, *n* (%)	19 (54.29)	27 (77.14)			

NPRP	No, *n* (%)	33.00 (94.29)	29 (82.86)			0.133
	Yes, *n* (%)	2.00 (5.71)	6 (17.14)			

*Note:* Chi‐square test; exact test, independent samples *t* test; *α*: 0.05; MD (CI), mean difference (95% confidence interval); ES (CI), effect size (95% confidence interval); NPRP, number of patients requiring postoperative additional dose of paracetamol; ARPO, analgesic requirement at postoperative. ^∗^Statistically significance *p* < 0.05.

Abbreviations: ASA = American Society of Anesthesiologists, BMI = body mass index.

In the postoperative pain assessment, no statistically significant difference was observed between the study groups in terms of VAS scores at 5, 30, and 60 min and at 3, 6, 12, and 24 h hours (*p* = 0.504 [effect size (95% CI): −0.20 (−0.66, 0.28)], *p* = 0.105 [effect size (95% CI): −0.39 (−0.86, 0.09)]), *p* = 0.607 [effect size (95% CI): −0.12 (−0.59, 0.35)], *p* = 0.688 [effect size (95% CI): 0.09 (−0.38, 0.56)], *p* = 0.226 [effect size (95% CI): −0.29 (−0.76, 0.18)], *p* = 0.948 [effect size (95% CI): 0.02 (−0.45, 0.48)], *p* = 0.889 [effect size (95% CI): 0.03 (−0.44, 0.50)], respectively). A preliminary investigation of preoperative IL‐6 and PGE2 levels revealed no statistically significant differences (*p* = 0.078 [effect size (95% CI): −0.43 (−0.90, 0.05)], *p* = 0.294 [effect size (95% CI): −0.25 (−0.72, 0.22)]). The acupuncture group demonstrated a reduction in postoperative IL‐6 levels (*p* = 0.014 [effect size (95% CI): −0.60 (−1.08, −0.12)]). Subsequent analysis revealed no statistically significant difference in postoperative PGE2 levels between the acupuncture and control groups (*p* = 0.568 [effect size (95% CI): −0.14 (−0.60, 0.33)]) (Table 2). Subsequent analyses revealed no significant differences between the groups in terms of postoperative nausea/vomiting (PONV) at 5–30–60 minutes and 3–6–12–24 hours (*p* = 0.232, *p* = 0.721, *p* = 0.495, *p* = 0.690, *p* = 0.232, *p* = 0.151, *p* = 0.151) (Table 3).

**Table 2:** Comparison of postoperative VAS scores, IL‐6, and PGE2 levels between acupuncture and control groups.

	Acupuncture Mean ± SD	Control Mean ± SD	MD (CI)	ES (CI)	*p*
VAS 5 min	3.59 ± 1.71	3.96 ± 2.05	−0.37 (−1.48, 0.74)	−0.20 (−0.66, 0.28)	0.504
VAS 30 min	5.17 ± 1.95	5.91 ± 1.84	−0.74 (−1.64, 0.16)	−0.39 (−0.86, 0.09)	0.105
VAS 60 min	3.83 ± 1.44	4.03 ± 1.77	−0.20 (−0.97, 0.57)	−0.12 (−0.59, 0.35)	0.607
VAS 3 h	3.34 ± 1.53	3.20 ± 1.43	0.14 (−0.56, 0.85)	0.09 (−0.38, 0.56)	0.688
VAS 6 h	3.26 ± 1.40	3.69 ± 1.53	−0.43 (−1.13, 0.27)	−0.29 (−0.76, 0.18)	0.226
VAS 12 h	3.77 ± 2.06	3.74 ± 1.58	0.03 (−0.85, 0.90)	0.02 (−0.45, 0.48)	0.948
VAS 24 h	2.94 ± 1.75	2.89 ± 1.66	0.06 (−0.76, 0.87)	0.03 (−0.44, 0.50)	0.889
PR PGE2 (pg/mL)	2846.84 ± 1146.92	3184.25 ± 1498.18	−337.41 (−973.81, 298.99)	−0.25 (−0.72, 0.22)	0.294
PO PGE2	2787.58 ± 1471.84	3036.77 ± 2104.39	−249.18 (−1117.22, 618.85)	−0.14 (−0.60, 0.33)	0.568
PR IL‐6 (pg/mL)	25.83 ± 5.43	29.64 ± 11.39	−3.81 (−8.07, 0.44)	−0.43 (−0.90, 0.05)	0.078
PO IL‐6	24.44 ± 4.57	30.21 ± 12.70	−5.77 (10.32, −1.21)	−0.60 (−1.08, −0.12)	0.014^**∗**^

*Note:* Independent samples *t* test; paired *t* test; *α*: 0.05; MD (CI), mean difference (95% confidence interval); ES (CI), effect size (95% confidence interval); min, minute; h, hours; PR, preoperative; PO, postoperative; PGE2, prostaglandin E2; IL‐6, interleukin‐6. ^∗^Statistically significance *p* < 0.05.

Abbreviation: VAS = visual analog scale.

**Table 3:** Postoperative nausea/vomiting comparison between acupuncture and control groups.

	Nause/vomiting	Acupuncture	Control	*p*
5 min	No, *n* (%)	33 (94.3)	30 (85.7)	0.232
	Yes, *n* (%)	2 (5.7)	5 (14.3)	

30 min	No, *n* (%)	31 (88.6)	30 (85.7)	0.721
	Yes, *n* (%)	4 (11.4)	5 (14.3)	

60 min	No, *n* (%)	29 (82.9)	31 (88.6)	0.495
	Yes, *n* (%)	6 (17.1)	4 (11.4)	

3 h	No, *n* (%)	31 (88.6)	32 (91.4)	0.690
	Yes, *n* (%)	4 (11.4)	3 (8.6)	

6 h	No, *n* (%)	33 (94.3)	30 (85.7)	0.232
	Yes, *n* (%)	2 (5.7)	5 (14.3)	

12 h	No, *n* (%)	35 (100.0)	33 (94.3)	0.151
	Yes, *n* (%)	0 0.0	2 (5.7)	

24 h	No, *n* (%)	35 (100.0)	33 (94.3)	0.151
	Yes, *n* (%)	0 0.0	2 (5.7)	

*Note:* Chi‐square test; exact test; *α*: 0.0; min, minute; h, hours.

Intraoperatively, the acupuncture group exhibited significantly lower DBP and MAP at 30 min and 60 min compared to the control group (*p* = 0.012, *p* = 0.026, *p* = 0.012, *p* = 0.012, respectively). Furthermore, at the 15th minute of the operation, the heart rate (HR) exhibited a higher value in the experimental group when compared to the acupuncture group (*p* = 0.039), as illustrated in Table 4. The acupuncture group had lower DBP at 5 min and significantly lower MAP 6 h after surgery (Table 5). The adverse events related to acupuncture were not assessed.

**Table 4:** Intraoperative hemodynamic comparison between acupuncture and control groups.

		SBP	DBP	MAP	HR	SpO_2_
		Mean ± SD	Mean ± SD	Mean ± SD	Mean ± SD	Mean ± SD
5 min	Acupuncture	127.49 ± 17.68	81.29 ± 16.95	98.06 ± 16.83	91.37 ± 13.77	98.91 ± 3.16
	Control	123.71 ± 18.39	78.83 ± 17.11	95.38 ± 16.07	90.86 ± 14.03	99.71 ± 2.57
	*p*	0.385	0.548	0.502	0.877	0.249

15 min	Acupuncture	113.31 ± 18.83	68.31 ± 16.72	86.89 ± 18.27	90.54 ± 15.56	99.37 ± 0.49
	Control	115.91 ± 15.44	71.46 ± 13.08	89.31 ± 14.93	83.69 ± 11.34	99.34 ± 0.64
	*p*	0.530	0.384	0.545	0.039^**∗**^	0.834

30 min	Acupuncture	105.83 ± 16.25	58.97 ± 13.07	77.69 ± 13.53	83.29 ± 14.30	99.46 ± 0.51
	Control	111.37 ± 15.28	67.60 ± 14.75	85.71 ± 15.94	80.14 ± 10.36	99.40 ± 0.60
	*p*	0.146	0.012^**∗**^	0.026^**∗**^	0.296	0.669

60 min	Acupuncture	108.83 ± 12.86	61.43 ± 11.31	81.49 ± 11.72	81.26 ± 15.40	99.37 ± 0.49
	Control	112.91 ± 16.87	69.20 ± 13.70	84.43 ± 14.64	79.66 ± 13.92	99.34 ± 0.59
	*p*	0.259	0.012^**∗**^	0.356	0.650	0.826

120 min	Acupuncture	107.60 ± 8.11	61.95 ± 7.32	81.10 ± 6.21	80.65 ± 13.48	99.25 ± 0.44
	Control	109.18 ± 11.19	65.76 ± 11.29	82.82 ± 11.49	77.94 ± 11.48	99.26 ± 0.65
	*p*	0.623	0.224	0.566	0.519	0.942

*Note:* Independent samples *t* test; *α*: 0.05; SpO_2_, oxygen saturation; min, minute. ^∗^Statistically significance *p* < 0.05.

Abbreviations: DBP = diastolic blood pressure, HR = heart rate, MAP = mean arterial pressure, SBP = systolic blood pressure.

**Table 5:** Postoperative hemodynamic comparison between acupuncture and control groups.

		SBP	DBP	MAP	HR	SpO_2_
		Mean ± SD	Mean ± SD	Mean ± SD	Mean ± SD	Mean ± SD
5 min	Acupuncture	135.40 ± 18.45	78.51 ± 13.62	98.89 ± 16.45	88.31 ± 14.12	98.51 ± 3.94
	Control	130.94 ± 12.99	85.97 ± 15.21	103.57 ± 13.63	87.23 ± 17.00	98.91 ± 0.37
	*p*	0.251	**0.034**	0.199	0.772	0.551

30 min	Acupuncture	130.94 ± 16.66	71.11 ± 11.19	93.06 ± 11.65	83.71 ± 11.94	99.11 ± 0.68
	Control	129.23 ± 12.93	71.06 ± 11.19	93.97 ± 9.66	78.63 ± 13.86	98.97 ± 0.38
	*p*	0.632	0.983	0.722	0.105	0.280

60 min	Acupuncture	123.00 ± 13.09	71.89 ± 10.42	92.00 ± 11.06	78.63 ± 11.63	99.11 ± 0.68
	Control	121.83 ± 13.65	72.89 ± 9.55	93.29 ± 10.05	76.80 ± 15.04	98.91 ± 0.37
	*p*	0.715	0.677	0.613	0.571	0.130

3 h	Acupuncture	120.03 ± 10.92	67.46 ± 7.02	91.00 ± 10.03	82.97 ± 13.66	99.03 ± 0.79
	Control	119.37 ± 10.93	70.03 ± 8.17	93.06 ± 8.93	80.54 ± 14.64	98.86 ± 0.60
	*p*	0.802	0.162	0.371	0.475	0.309

6 h	Acupuncture	114.77 ± 9.64	67.57 ± 8.59	88.06 ± 8.22	85.57 ± 12.09	99.03 ± 0.79
	Control	115.77 ± 10.76	68.77 ± 7.87	92.31 ± 8.85	82.34 ± 13.14	98.94 ± 0.59
	*p*	0.683	0.544	**0.041**	0.289	0.608

12 h	Acupuncture	111.23 ± 20.15	66.43 ± 8.31	87.66 ± 7.93	83.91 ± 11.35	99.06 ± 0.64
	Control	113.97 ± 13.50	68.57 ± 7.70	91.23 ± 7.28	80.09 ± 12.70	98.97 ± 0.45
	*p*	0.506	0.267	0.054	0.188	0.520

24 h	Acupuncture	117.69 ± 11.12	70.83 ± 10.29	90.17 ± 9.94	85.26 ± 10.14	99.09 ± 0.51
	Control	110.20 ± 23.32	70.17 ± 9.96	90.89 ± 9.60	81.31 ± 11.71	99.09 ± 0.28
	*p*	0.091	0.787	0.761	0.137	1.000

*Note:* Independent samples *t* test; *α*: 0.05; SpO_2_, oxygen saturation; min, minute; h, hours. Bold values represent statistically significant values (*p* < 0.05).

Abbreviations: DBP = diastolic blood pressure, HR = heart rate, MAP = mean arterial pressure, SBP = systolic blood pressure.

### 3.1. Side Effects

Patients received PNs 24 h before surgery. These were checked for proper positioning in the preoperative and postoperative patient rooms. A total of four acupuncture points were stimulated. No adverse effects were observed.

## 4. Discussion

Although there was no statistically significant difference in VAS scores between the acupuncture group and the control group, the study showed that patients in the acupuncture group required fewer analgesics 30 min after septorhinoplasty surgeries. Additionally, the investigation revealed that the IL‐6 levels at 24 h postoperatively were lower in the acupuncture group. Acupuncture was ineffective for preventing PONV in septorhinoplasty surgeries. Intraoperatively, DBP and HR were lower at 30 min and DBP was lower at 60 min, whereas HR was higher at 15 min. Conversely, in the postoperative period, both the DBP and MAP were lower at 5 min and 6 h, respectively.

According to the principles of traditional Chinese medicine, Neiguan (PC 6), Zusan Li (ST 36), and Hegu (LI 4) are considered to be of significant importance for the purpose of achieving analgesia through acupuncture [22].

The study by Wang et al. demonstrated that the Neiguan (PC 6), Zusan Li (ST 36), and Hegu (LI 4) acupoints significantly reduced intraoperative remifentanil usage and postoperative side effects in patients undergoing sinusotomy [23]. It has been reported that acupuncture points including LI 4 and PC 6 effectively maintain analgesia and hemodynamic stability during thyroidectomy operations [24]. As Sim et al. demonstrated, preoperative administration of acupuncture to ST 36 and PC 6 points led to a reduction in intraoperative and postoperative opioid utilization [25]. In the present study, a reduced necessity for analgesics was observed in the acupuncture group at 30 min following septorhinoplasty surgery. The control group exhibited higher VAS values; however, this difference was not statistically significant. A meta‐analysis of studies combining acupuncture and drug treatment showed that acupuncture to the sphenopalatine ganglion reduced the VAS score [26]. A systematic review and meta‐analysis of acupuncture point stimulation concluded that there is insufficient evidence to demonstrate its effectiveness in managing postoperative pain in surgical patients [27]. A meta‐analysis study conducted by Deng et al. showed that, compared to the sham acupuncture group or control group, the combination of acupuncture with patient‐controlled analgesia reduced pain intensity and opioid dosage after back surgery. However, the incidence of PONV was not significantly different [28]. Conversely, Lindsey et al. demonstrated that acupuncture following total knee arthroplasty surgery reduced the incidence of PONV. However, it did not reduce VAS scores within the first 48 h after surgery, but did reduce them after this time [29]. The type of acupuncture used alongside surgery, as well as the acupuncture points stimulated, can alter the effect of acupuncture on postoperative pain. The level of postoperative pain experienced for each surgical procedure, as well as the effect of each acupuncture point on pain, may vary.

In the context of surgical inflammation, the serum levels of IL‐6 exhibit an increase within the first hour to 3 hours postsurgery, reaching a peak between 4 and 24 h after the procedure. Thereafter, IL‐6 levels remain elevated for a duration of 48–72 h [30]. Due to financial limitations, our study was constrained to analyzing IL‐6 levels at the 24‐h mark postoperatively. The acupuncture group exhibited lower levels of IL‐6 in comparison to the control group. A study demonstrated that electroacupuncture (EA) stimulation suppressed inflammatory cytokines and IL‐6 in cases of posttraumatic spinal damage. EA stimulation has been applied at the GV 6 (Jizhong) and GV 9 (Zhiyang) points [31]. Ertha et al. demonstrated that the application of acupuncture at the ST 36 point resulted in a reduction of pain‐related behaviors in rats [32]. In a study on septoplasty surgeries, Sahmeddini et al. used LI 11 (Quchi), HT 7 (Shenmen), LI 4 (Hegu), and PC 6 (Neiguan) acupuncture points for postoperative pain management. In summary, the findings indicated that the combination of EA and morphine led to comparable postoperative pain scores and analgesic utilization. Furthermore, EA demonstrated an analgesic effect that was analogous to that of morphine [6].

Huili et al. investigated the impact of acupuncture on spinal PGE2 expression in the context of EA at “distal acupuncture + proximal acupuncture” points. Their findings indicated that EA possesses the capacity to modulate PGE2 expression within the spinal cord. The EX‐B2 (Jiaji), BL 25 (Dachangshu), BL 40 (Weizhong), and BL 60 (Kunlun) points were used for rats in bilateral EA Group 1; the BL 40 (Weizhong) and BL 60 (Kunlun) points in EA Group 2; and the EX‐B2 (Jiaji) and BL 25 (Dachangshu) points in EA Group 3. The results demonstrated that the upward regulation of spinal PGE2 levels was reversed in groups EA 1, 2, and 3 compared to the control group. The investigation revealed no statistically significant differences in spinal PGE2 levels among the three groups: EA 1, EA 2, and EA 3. The integration of “distal acupuncture and proximal acupuncture points” has gained significant traction in clinical settings [33]. In their study, Jiang et al. demonstrated that EA alleviates labor pain in rats by inhibiting the release of PGE2 via the p38 MAPK pathway in the spinal cord and reducing PGE2 expression in the uterus [34]. In contrast to the aforementioned study, our investigation employed PC 6 and ST 36 points. The absence of a change in PGE2 in our study may be attributable to the utilization of disparate acupuncture points.

Acupressure at the PC 6 acupuncture point has been demonstrated to have a significant impact on the reduction of PONV in patients undergoing gynecological surgery, laparoscopic cholecystectomy, and appendectomy, as reported in numerous studies [35–37]. Contrary to these findings, a separate study revealed that acupressure administered at PC 6 was ineffective in mitigating PONV [38]. In our study, acupuncture demonstrated no impact on symptoms of nausea and vomiting.

As demonstrated by Ma et al., the administration of acupuncture 30 min prior to the induction of anesthesia resulted in a significantly more stable HR and MAP in the acupuncture group. Additionally, in the acupuncture group, adrenal cortical inhibition and decreased catecholamine secretion during surgery were observed [39]. In the study by Wu et al., perioperative stress responses in craniotomy were compared, and it was demonstrated that the EA group exhibited a more stable HR and arterial pressure, as well as significantly lower levels of epinephrine, cortisol, and blood sugar [40]. Another study demonstrated that the application of acupuncture at the LI 4 (Hegu) and PC 6 (Neiguan) points during intravenous anesthesia in thyroidectomy surgeries resulted in a more stable MAP in the acupuncture group [41]. Conversely, Yang et al. observed no statistically significant alterations in HR in their study [42]. The findings of our study indicated that, intraoperatively, DBP and HR exhibited a decline at the 30‐min mark, with DBP demonstrating a further decrease at the 60‐min interval. Concurrently, HR showed an increase at the 15 min point. Postoperatively, both the DBP and the MAP exhibited a decrease at 5 min and 6 h, respectively. The study’s findings were not entirely conclusive owing to the absence of the bispectral index (BIS) in the analysis owing to budgetary constraints.

The effect of sham acupuncture is inconsistent compared to real acupuncture [43]. For this reason, a sham group was not included in the study.

### 4.1. Limitations

The single‐center nature of the study, in conjunction with the fact that acupuncture was administered while the patient was awake and conscious, may have influenced the observed outcomes, particularly the analgesic consumption in the acupuncture group. In fact, although there was a reduction in analgesic consumption in our study, there was no statistically significant difference in VAS scores. The present study did not investigate the mechanisms of PN acupuncture pretreatment in humans. In order to circumvent the impact of intraoperative factors on postoperative analgesic requirements, the utilization of BIS for the monitoring and standardization of general anesthesia in future studies is recommended. The investigation of IL‐6 and PGE2 levels in the postoperative period could have been conducted at 6–12 h or at various other time points. The findings of this study indicate that PN acupuncture holds promise as a viable strategy in septorhinoplasty cases, owing to its cost‐effectiveness, the simplicity of pretreatment, and the ease of application. A multicenter study with a large sample size is necessary to address these limitations.

## 5. Conclusion

To the best of our knowledge, this is the first randomized clinical trial investigating the effects of acupuncture in septorhinoplasty surgeries. Although there was no statistically significant difference in VAS scores between the acupuncture group and the control group, our study showed that the acupuncture group required fewer analgesics 30 min after surgery and had lower IL‐6 levels 24 h after surgery.

## Conflicts of Interest

The authors declare no conflicts of interest.

## Author Contributions

Yavuz Orak: conceptualization, investigation, and writing. Filiz Alkan Baylan: study of biochemical parameters and investigation. Fatma Bilgen: conceptualization and data collection. Filiz Orak: investigation, conceptualization, and writing. Alper Ural: conceptualization and data collection. Sedat Yildiz: conceptualization and writing. Saime Sagiroglu: conceptualization and data collection. Harun Karaduman: writing and editing. Adem Doganer: data analysis.

## Funding

This research did not receive any specific grant from funding agencies in the public, commercial, or not‐for‐profit sectors.

## Data Availability

The readers can contact the authors if further information is required.
